# Antiangiogenic and Antitumor Effects of *Trypanosoma cruzi* Calreticulin

**DOI:** 10.1371/journal.pntd.0000730

**Published:** 2010-07-06

**Authors:** Nandy C. López, Carolina Valck, Galia Ramírez, Margarita Rodríguez, Carolina Ribeiro, Juana Orellana, Ismael Maldonado, Adriana Albini, Daniel Anacona, David Lemus, Lorena Aguilar, Wilhelm Schwaeble, Arturo Ferreira

**Affiliations:** 1 Institute of Biomedical Sciences, Faculty of Medicine, University of Chile, Santiago, Chile; 2 Oncology Research, Science and Technology Pole, IRCCS Multimedica, Milan, Italy; 3 Department of Infection, Immunity and Inflammation, University of Leicester, Leicester, United Kingdom; New York University School of Medicine, United States of America

## Abstract

**Background:**

In Latin America, 18 million people are infected with *Trypanosoma cruzi*, the agent of Chagas' disease, with the greatest economic burden. Vertebrate calreticulins (CRT) are multifunctional, intra- and extracellular proteins. In the endoplasmic reticulum (ER) they bind calcium and act as chaperones. Since human CRT (HuCRT) is antiangiogenic and suppresses tumor growth, the presence of these functions in the parasite orthologue may have consequences in the host/parasite interaction. Previously, we have cloned and expressed *T. cruzi* calreticulin (TcCRT) and shown that TcCRT, translocated from the ER to the area of trypomastigote flagellum emergence, promotes infectivity, inactivates the complement system and inhibits angiogenesis in the chorioallantoid chicken egg membrane. Most likely, derived from these properties, TcCRT displays *in vivo* inhibitory effects against an experimental mammary tumor.

**Methodology and Principal Findings:**

TcCRT (or its N-terminal vasostatin-like domain, N-TcCRT) a) Abrogates capillary growth in the *ex vivo* rat aortic ring assay, b) Inhibits capillary morphogenesis in a human umbilical vein endothelial cell (HUVEC) assay, c) Inhibits migration and proliferation of HUVECs and the human endothelial cell line Eahy926. In these assays TcCRT was more effective, in molar terms, than HuCRT: d) In confocal microscopy, live HUVECs and EAhy926 cells, are recognized by FITC-TcCRT, followed by its internalization and accumulation around the host cell nuclei, a phenomenon that is abrogated by Fucoidin, a specific scavenger receptor ligand and, e) Inhibits *in vivo* the growth of the murine mammary TA3 MTXR tumor cell line.

**Conclusions/Significance:**

We describe herein antiangiogenic and antitumor properties of a parasite chaperone molecule, specifically TcCRT. Perhaps, by virtue of its capacity to inhibit angiogenesis (and the complement system), TcCRT is anti-inflammatory, thus impairing the antiparasite immune response. The TcCRT antiangiogenic effect could also explain, at least partially, the *in vivo* antitumor effects reported herein and the reports proposing antitumor properties for *T. cruzi* infection.

## Author Summary

In Latin America, 18 million people are infected with *Trypanosoma cruzi*, a protozoan that causes Chagas' disease. Vertebrate calreticulins (CRTs) are multifunctional, intra- and extracellular calcium binding, chaperone proteins. Since human CRT (HuCRT) inhibits capillary growth (angiogenesis) and suppresses tumor growth, the presence of these functions in *T. cruzi* CRT (TcCRT) may have interesting consequences in the host/parasite interactions. Previously, we have cloned and expressed TcCRT and shown that, when translocated from the endoplasmic reticulum to the area of trypomastigote flagellum emergence, it promotes infectivity, inactivates the complement system, an innate defense arm and inhibits angiogenesis in the chorioallantoid chicken egg membrane. TcCRT inhibits angiogenesis, since it interferes with endothelial cell multiplication, migration and capillary morphogenesis *in vitro*, as well as angiogenesis in rat aortic rings. The parasite molecule also displays important antitumor effects. In these activities, TcCRT is more effective than the human counterpart. Perhaps, by virtue of its capacity to inhibit angiogenesis, TcCRT is anti-inflammatory, thus impairing the antiparasite immune response. The TcCRT antiangiogenic effect could also explain, at least partially, the *in vivo* antitumor effects reported herein and the reports proposing antitumor properties for *T. cruzi* infection.

## Introduction

Chagas′ disease affects 16 million people in South America, with 14.000 deaths per year and 0.7 million disability-adjusted life-years [1]. *T. cruzi* has a variety of molecules that modulate several effector arms of the immune system [2], calreticulin (TcCRT) being one of them [3]. TcCRT, first isolated in our laboratory [4], [5], is highly homologous with human calreticulin (HuCRT) [6], an exceedingly pleiotropic chaperone molecule [7]. In spite of its primary endoplasmic reticulum (ER) location, TcCRT is also expressed on the cell membrane [3].

Based on their capacity to bind laminin [8] and to inhibit endothelial cell proliferation, both HuCRT and its N-terminal fragment, vasostatin or N-TcCRT, display antiangiogenic properties *in vitro* and *in vivo* [9], [10]. These HuCRT properties are paralleled by inhibitory activities on several tumor models [11]–[13]. Identifying these properties in TcCRT may define important aspects of the host/parasite interaction.

We have recently reported that TcCRT is strongly antiangiogenic in the chorioallantoid membrane in chicken eggs (CAM assay) [14]. Since angiogenesis modulators behave differently across species [15], we verified this effect in different experimental set ups in mammals, *Homo sapiens sapiens* included. Thus, TcCRT and its vasostatin-like domain, inhibit angiogenesis in the *ex vivo* rat aortic ring assay. It also affects key cellular angiogenic parameters in human endothelial cell cultures, such as proliferation, chemotaxis and cell morphogenesis into tubular-like structures in Matrigel. These results correlate with TcCRT binding and internalization in these cells. Perhaps, the TcCRT antiangiogenic (and anti-complement) properties result in anti inflammatory outcomes, thus inhibiting the host antiparasite immune response. Also, at least a partial explanation for those reports [16], [17] proposing anti-tumor effects for trypanosome infection is herein provided. Although anti-tumor effects have been reported for several decades now, for a variety of infections with other microbial agents [18], [19], pathogen molecules mediating those statistically based tumor resistances, have been poorly defined. In synthesis, here we describe that a parasite chaperone molecule, most likely by interacting with endothelial cells, and inhibiting angiogenesis, interferes with tumor growth.

## Methods

### Ethics statement

Human umbilical vein endothelial cells (HUVECs) were isolated [20], following informed patient's written consent (University of Chile Hospital Bioethics Committee).

### Cells

The human endothelial EAhy926 cell line (kindly provided by Dr. Gareth Owen, Pontifical Catholic University, Chile), was maintained in Iscove's Modified Dulbecco's Medium (IMDM, Invitrogen, USA) with 10% fetal bovine serum (FBS, Invitrogen, USA) and 100 units/ml penicillin/streptomycin (Sigma, USA). HUVECs were 80% pure by flow cytometry and immunofluorescence using anti CD31 monoclonal antibodies (Sigma, USA) as a marker. The cells were cultured in M199 medium (Sigma, USA), with 20% FBS, 2 mM glutamine (Invitrogen, USA), 100 units/ml penicillin/streptomycin, 100 µg/ml endothelial cell growth supplement (ECGS) (BD Biosciences, USA), and 10 µg/ml heparin (Sigma, USA) in gelatin-coated flasks.

### Recombinant proteins

TcCRT, its R-domain (R-TcCRT) and HuCRT were obtained from *E. coli* [3], [21]. N-TcCRT (amino acids 20–193, GenBank accession no. **AF162779**) was amplified by PCR using Tli DNA polymerase (Promega, USA). Primers were: (5′-GGAATTCCACGGTGTACTTCCACGAG-3′) and (5′- CTCGAGCCAGTCTTCTTCGAGCTG-3′).

N-TcCRT DNA was ligated into the *Eco*RI and XhoI sites of the pET-28b (+) plasmid (Novagen, UK). Competent *E. coli* TOP10F′ bacteria were transformed, plated and selected with 50 µg/ml ampicillin. *E. coli* BL21 (DE3)pLysS was transformed with the plasmid and grown in the presence of 34 µg/ml chloramphenicol with 50 µg/ml kanamycin. After adding isopropyl β-D-thiogalactoside and 3 h incubation, the cells were sonicated, centrifuged, and the supernatants filtered. The recombinant proteins were purified using His Bind resin (Novagen, UK), eluted with buffer containing 1 M imidazole, and dialyzed against 2 mM Tris-HCl and 150 mM NaCl, pH 7.4. Both, TcCRT and N-TcCRT were tested for endotoxin by the Limulus Amebocyte Lysate Kinetic-QCL assay (BioWhittaker, USA) and contained <5 EU/10 mg protein.

The R-TcCRT domain (aa 136–281) was expressed and purified as previously described [3].

### Rat aortic ring assay

This *ex vivo* angiogenesis assay [22], was performed with slight modifications. Six week old Sprague-Dawley rats, from our Animal Facility were used in this experiment. Briefly, the animals were sacrificed by CO_2_ inhalation, their thoracic aortas dissected and sliced into 1 mm thick rings. Two or three rings per well were placed on a 24-well plate and embedded in 100 µl Matrigel (BD Biosciences, USA), followed by 30 min incubation. Wells were overlaid with 300 µl of FBS-supplemented M199 medium with 100 µg/ml ECGS and phosphate buffered saline (PBS) or several TcCRT concentrations. The rings were incubated for 7 days and visualized under phase contrast in a Nikon Eclipse E400 microscope. Fields were photographed and the length of capillaries measured using Adobe Photoshop software. For each experiment and in sextuplicate, 3 capillaries (shortest, medium and longest) per ring were measured. The average length was considered as 100%. The statistical validation of these experiments was defined by the Student's t-test.

### Matrigel morphogenesis assay

24-microwell plates were filled with 300 µl Matrigel/well and polymerized for 1 h at 37°C. 70×10^3^ HUVECs/well were suspended in FBS-supplemented M199 medium, with 100 µg/ml ECGS and several TcCRT, N-TcCRT, lypopolisaccharide (LPS), HuCRT or R-TcCRT concentrations. The cells were layered on the gel. After 6 h incubation, morphogenesis was assessed by phase contrast microscopy and images were imported into the Adobe Photoshop program. Tubular capillary-like structures were quantified by manual counting in 40× fields, in quadruplicates, as previously described [23]. Data were analyzed by one way ANOVA. Values are reported as means ± SEM. Comparison of means was performed by the Bonferroni method.

### Chemotaxis assay

With HUVECs, the assays were performed in Boyden chambers, while Transwell chambers (Costar, USA) were used with EAhy926 cells [24]. HUVECs were pretreated for 24 h with PBS, LPS, or variable TcCRT concentrations in FBS-supplemented M199 medium. EAhy926 cells were pretreated with IMDM containing several TcCRT concentrations. 7.5×10^4^ HUVECs or 5×10^4^ EAhy926 cells/chamber were washed, resuspended in serum-free medium, and placed in the upper compartment, with or without TcCRT or LPS. Supernatants from NIH3T3 cells (for HUVECs) or 10% FBS (for EAhy926) were used as chemo attractants in the lower chamber. After 6 h (HUVECs) or 16 h (EAhy926) incubation, the cells on the upper filter surface were removed, and those on the lower surface, fixed and stained. Filters were photographed with CCD optics and a digital analysis system (Image ProPlus, Media Cybernetics, Silver Spring, MD) and nine fields per filter were counted (HUVECs). EAhy926 cell migration was measured by densitometry analysis at 595 nm. All experiments were performed in triplicates. Data were analyzed by one way ANOVA. Values are reported as means ± SEM. Comparison of means was performed by the Bonferroni method.

### Proliferation assays

These assays were quantified using MTT (3-[4,5-dimethylthiazol-2-yl]2,5-diphenyltetrazoliumbromide, Calbiochem, USA) or crystal violet reagents. Briefly, in the MTT assay, 2,500 HUVECs/well were seeded in sestuplicate in 96-well plate and growth, in the presence of various TcCRT, N-TcCRT or HuCRT concentrations, was assessed at 24-h periods over 4 days. Then, MTT was added, incubated for 4.5 h, solubilized in DMSO and the absorbance was read at 550 nm. The same assay was performed with 2,000 VERO cells, as a negative control showing that recombinant TcCRT did not affect the *in vitro* growth of an unrelated cell line. Data were analyzed by one way ANOVA, followed by the Bonferroni test. Values are reported as means ± SEM. In the crystal violet assay, the same number of HUVECs were seeded in gelatin-coated wells and treated with R-TcCRT at different concentrations. The number of viable cells was measured over time with the crystal violet reagent, following standard procedures.

### Protein binding and internalization assays

TcCRT was labeled with the FluoReporter FITC Protein Labeling Kit (Molecular Probes, USA). HUVECs or EAhy926 cells were incubated with 1 µM TcCRT, FITC-TcCRT or FITC-TcCRT plus 10 µM unlabelled TcCRT, for 1 h. After washing, the cells were fixed with 4% paraformaldehyde, for 15 min at room temperature, washed and mounted in 50% glycerol, containing 4′-6-diamidino-2-phenylindole (DAPI). Slides were visualized in a Nikon Eclipse E400 epifluorescence microscope. Protein uptake was detected by incubating the cells for 30 min, in medium containing 1 µM FITC-TcCRT, alone or in the presence of 25 µg/ml fucoidin (Sigma, USA). Images were collected using the LSM510 Software system attached to a Zeiss (Oberkochen, Germany) LSM510meta confocal microscope.

### Tumor growth assay

The TcCRT and HuCRT effects on *in vivo* growth of the TA3 MTXR murine mammary tumor cell line was assessed in 2 independent experiments, performed 6 months apart, in adult female A/J mice. Four animals were used in the first experiment and 6 in the second one. In both experiments, the animals were inoculated s.c., every other day, with 50 µg TcCRT or HuCRT or solvent, during 25 days. At day 0, the animals were challenged with 5×10^5^ tumor cells. Tumor size was determined with a digital caliper (Mitutoyo Corp, Japan), in a double blind procedure, as previously described [25]. The experiments were validated by using the Wilcoxon Signed Rank test (GraphPad Prism 4). P values≤0.05 were considered as statistically significant.

### Animal welfare

Six week old New Zealand rats and adult (20–25 g) female A/J mice were obtained from our Central Animal Facility. Experiments were performed in compliance with the “Guide for the Care and Use of Laboratory Animals”, National Research Council, Washington DC, USA, 2002. All procedures with these animals were approved by the local Bioethics Committee (Bioethics Committee, Faculty of Medicine, University of Chile). Surgeries and sacrifices were performed by the Animal Facility Veterinary Surgeons.

## Results

### TcCRT inhibits angiogenesis in the rat aortic ring assay

**Figure 1:**
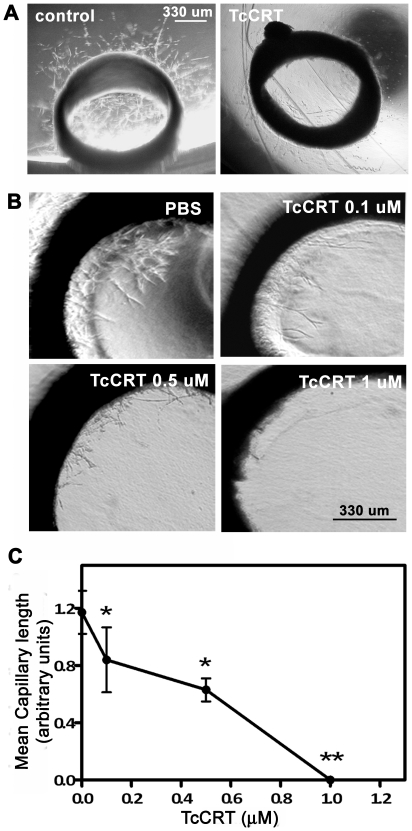
TcCRT inhibits angiogenesis in an *ex vivo* assay. Aortic rings were embedded in Matrigel and incubated in supplemented media at 37°C, 5% CO_2_, for 7 days. **A**. Representative images of aortic rings normal capillary sprouting (control) and in response to 1 µM TcCRT. **B**. Dose-dependent TcCRT inhibitory effect on angiogenesis. **C**. Quantitative analysis of the inner ring vessel length shown in B. Data are shown as means ± SEM, obtained from individual rings and are representative of at least 3 rings in each experiment and two independent experiments. *, p<0.05. **, p<0.01. Original magnification, ×4.

Two representative experiments are shown in Figure 1, A–B. Micro vessels are observed after culturing the aortic rings for 1 week (Figure 1A, control). Incubation with 1 µM TcCRT mediated complete capillary growth abrogation (Figure 1A, TcCRT). A dose-dependent antiangiogenic effect is observed (Figure 1B), until reaching complete capillary growth arrest. In Figure 1C, quantification of this TcCRT inhibitory capacity is shown. At concentrations of 0.1 and 1.0 µM, about 50% and 100% inhibition is respectively observed. In separate experiments, the vasostatin like N-TcCRT also inhibits angiogenesis in this *ex vivo* experimental model (data not shown).

### TcCRT and its N-TcCRT inhibit endothelial cell capillary morphogenesis

**Figure 2:**
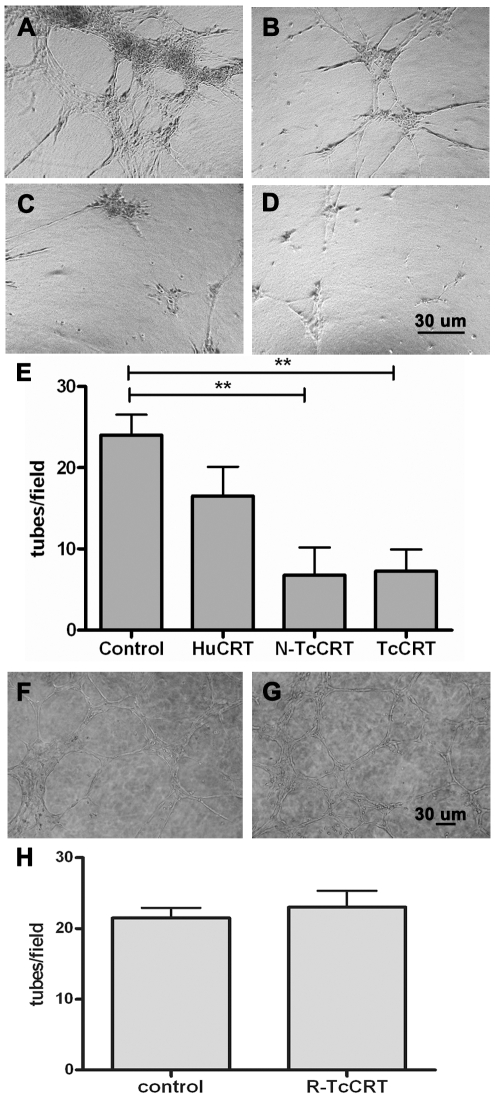
TcCRT and N-TcCRT inhibit capillary morphogenesis. Phase contrast images of HUVECs organization in the Matrigel morphogenesis assay are shown. Cells were cultured on the surface of Matrigel and incubated with: **A** and **F**. PBS (control), **B**. HuCRT, **C**. TcCRT, **D**. N-TcCRT and **G**. R-TcCRT, all of them at 1µM, for 6 h at 37°C, 5% CO_2_. **E** and **H**. Tubular structures were quantified by counting at low power fields. Data are represented as means ± SEM, obtained from four fields. **, p<0.01. Results are representative of 3 independent experiments. Original magnification, ×10.

A set of representative experiments is shown in Figure 2. In a 5-hour culture, control non-treated HUVECs generated a typical cell network (Figure 2A). Although strong inhibitory effects were observed with 1 µM HuCRT (Figure 2B), when N-TcCRT (Figure 2C) and TcCRT (Figure 2D) were compared at equal molarities with HuCRT, the effects of the parasite–derived molecules were clearly stronger than those of the human counterpart. Figure 2E shows the quantification of these assays. The TcCRT inhibitory effect was dose-dependent down to 0.1 µM (data not shown), while R-TcCRT did not affect capillary morphogenesis (Figure 2F–H).

### TcCRT inhibits endothelial cell migration

**Figure 3:**
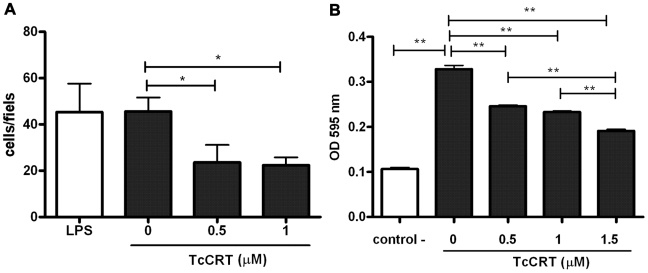
TcCRT inhibits human endothelial cell migration. **A**. HUVECs or **B**. EAhy926 cells migration towards chemo attractants was tested in the presence of increasing TcCRT or LPS concentrations. Serum-free medium was used as negative control. Experiments were performed in triplicates. Data are shown as means of values ± SEM obtained from triplicates of one representative experiment out of 3 independent ones. *, p<0.05. **, p<0.01.

HUVECs migration, as a response to the strong angiogenic factors present in NIH/3T3 cell conditioned media, was inhibited in a dose-dependent manner by TcCRT. LPS, at concentrations similar to those present in the TcCRT 1 µM preparation, showed no detectable effects (Figure 3A). Treatment with TcCRT also significantly inhibited migration of Eahy926 cells in response to FBS, over the same dose range (Figure 3B).

### TcCRT and N-TcCRT inhibit HUVEC proliferation

**Figure 4:**
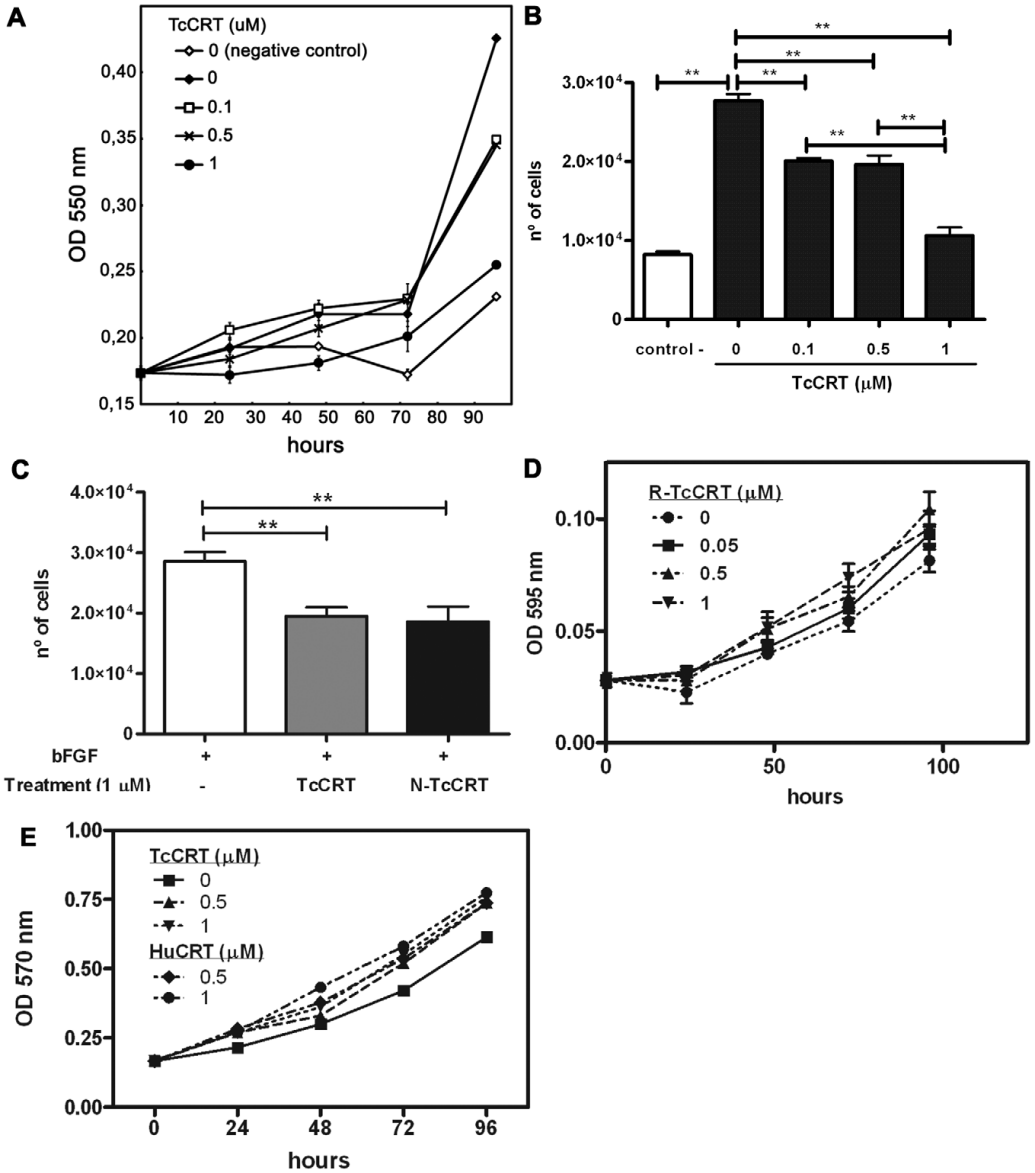
TcCRT inhibits HUVECs proliferation. **A**. Cells were grown in ECGS and FBS supplemented media, in the presence of various TcCRT concentrations. As negative control, the cells were grown in free growth factor and FBS media. Cell number was assessed at 24-hour periods over 4 days by the MTT method. **B**. Statistical analysis of the results shown in A, at 96 h. Data are representative of 3 independent experiments, performed in sestuplicate. **, p<0.01. **C**. Cells were grown in bFGF supplemented media, with or without 1 µM TcCRT or N-TcCRT. Cell number was determined after a 96-hour period by the MTT method. Data are shown as means ± SEM obtained from sestuplicate of one representative experiment. **, p<0.01. **D**. Cells were grown in ECGS and FBS supplemented media, in the presence of various R-TcCRT concentrations. Cell number was assessed at 24-hour periods over 4 days by de Cristal Violet method. Data are shown as means ± SEM from sestuplicates of one representative experiment. Non significant differences were obtained by ANOVA analysis. **E**. VERO cells were grown in FBS supplemented RPMI media, in the presence of various TcCRT or HuCRT concentrations. Cell number was assessed at 24-hour periods over 4 days by the MTT method. Data are shown as means ± SD obtained from sestuplicates of one representative experiment. Non significant differences were obtained by ANOVA analysis.

Figure 4 summarize these experiments. TcCRT inhibited endothelial cell proliferation in a dose-dependent manner, when they were stimulated with ECGS (Figure 4A). Maximum inhibition (60%) was observed with 1 µM TcCRT, at 96 hours (Figure 4B). A similar activity was also observed when TcCRT or N-TcCRT were added to HUVECs stimulated with basic fibroblast growth factor (bFGF) (Figure 4C). R-TcCRT, up to 1 µM, had no significant effects on HUVECs proliferation (Figure 4D). TcCRT did not affect VERO cell proliferation (Figure 4E), used as negative control.

### TcCRT binds to human endothelial cells and is internalized

**Figure 5:**
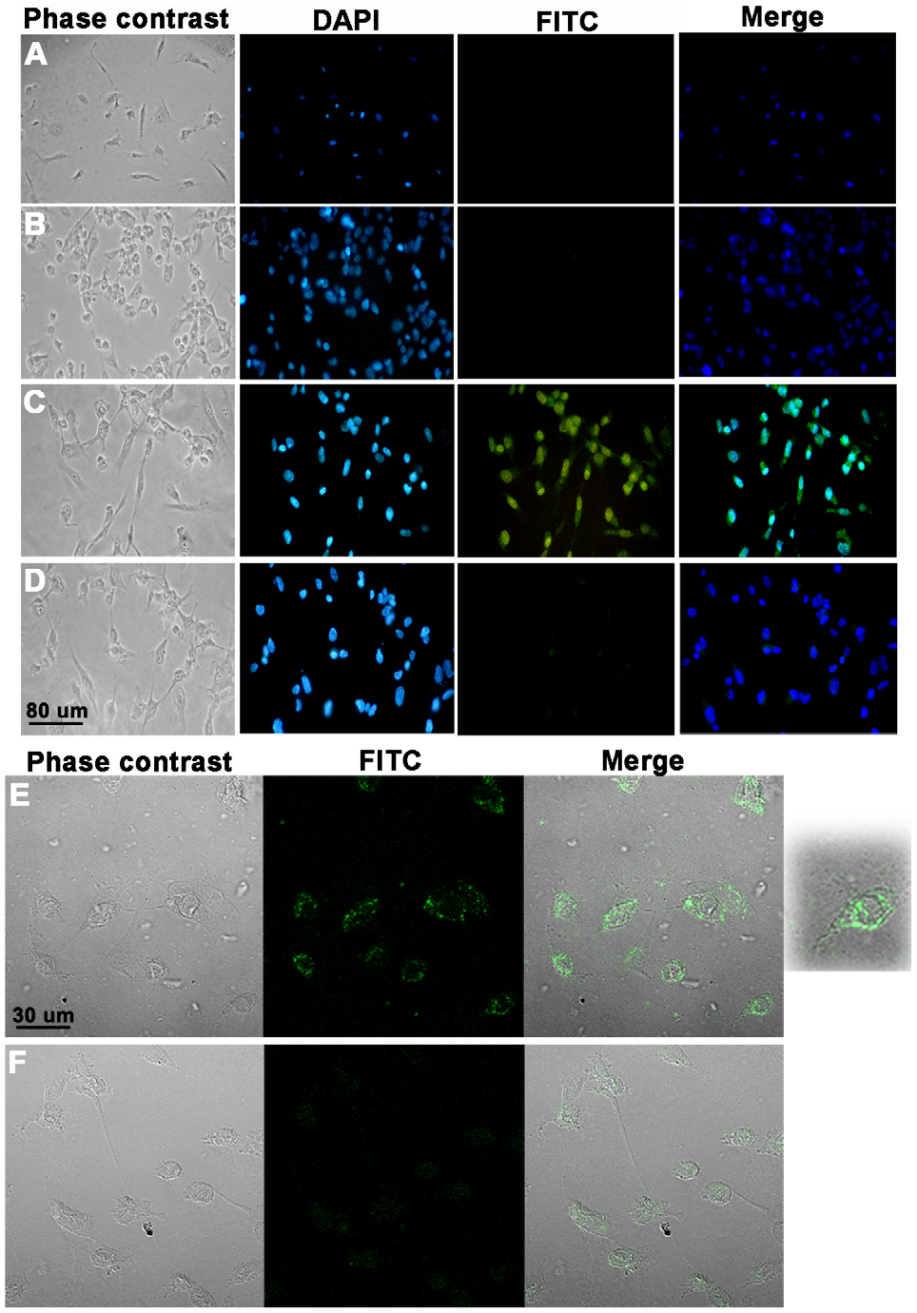
TcCRT binds to live HUVECs and is internalized. HUVECs were incubated with: **A**. FITC or 1 µM: **B**. TcCRT, **C**. TcCRT-FITC, **D**. TcCRT-FITC + 10 µM TcCRT, **E**. TcCRT-FITC and **F**. TcCRT-FITC + 25 µg/ml fucoidin, for 1h at 37°C, 5% CO_2_. The cells were then washed, fixed and analyzed by fluorescence (A–D) or confocal microscopy (E and F). Results are representative of three independent experiments. Original magnification, ×40 (A–D) and ×100 (E and F).

Although both HuCRT [8] and TcCRT bind to laminin, only the former interferes with the adhesion of endothelial cells to this molecule (data not shown). Therefore, the TcCRT antiangiogenic effect may be explained by other mechanisms, such as direct interaction with endothelial cells. FITC-TcCRT binds to live HUVECs (Figure 5C). This binding is reversed by a molar excess of the unlabeled protein (Figure 5D). Given the similarity between the DAPI and FITC-TcCRT mediated signals in this experiment (Figure 5C, merge), confocal microscopy was used to test if TcCRT was internalized after binding to the cell surface. After 30 min incubation, TcCRT accumulates around the HUVECs nuclei, in punctuate structures (Figure 5E), a phenomenon also observed in EAhy926 endothelial cells (data not shown). In order to better substantiate the TcCRT internalization by endothelial cells, an enlargement of a representative cell is shown (extreme right panel in Figure 5E). TcCRT internalization seems to be receptor-dependent, since fucoidin, a specific scavenger receptor ligand [26], [27], abrogated TcCRT uptake (Figure 5F).

### TcCRT inhibits the growth of a murine mammary tumor

**Figure 6:**
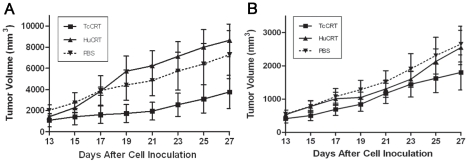
TcCRT inhibits the development of a murine A/J mammary tumor (TA3 MTXR). Two *in vivo* experiments, performed six months apart, are shown (A–B). 5×10^5^ tumor cells were inoculated s.c. in the 10 A/J female mice used in both experiments. TcCRT and HuCRT treatments, as well as measurement of tumor development, are described in the Methods section. TcCRT showed an anti-tumor effect in both experiments (p = 0.0078) and these effects were stronger (p = 0.0078) than those promoted by HuCRT. Bars represent standard errors.

The TcCRT and HuCRT effects on the *in vivo* growth of the TA3 MTXR murine A/J mammary tumor cell line was assessed in adult mice, in two independent experiments, performed 6 months apart (Figure 6A–B). Under the experimental conditions used, only the parasite chaperone molecule displayed significant (p = 0.0078) inhibitory effects on this tumor cell line, in both cases (Figure 6A–B). In one experiment (Figure 6A), TcCRT displayed a stronger antitumor effect, than the human orthologue (p = 0.0078 vs p = 0.1094). In the second experiment, HuCRT also had an effect (Figure 6B, p = 0.0078). However, again TcCRT had a stronger antitumor effect than HuCRT (p = 0.0078) (Figure 6B).

## Discussion

We have shown that TcCRT strongly inhibits capillary growth in the CAM *in vivo* assay [14]. Since angiogenesis modulators behave differently, not only across species, but also depending on the assay used [15], we studied the TcCRT antiangiogenic properties in the rat, a natural *T. cruzi* host. The *ex vivo* rat aortic ring assay provides a model closer to the physiologic *in vivo* situation, since endothelial cells are in a quiescent state, in a natural histological environment. In this assay, TcCRT completely abrogates capillary growth, in a dose-dependent manner (Figure 1). Capillary morphogenesis in Matrigel is a valid *in vitro* correlate of *in vivo* angiogenesis. As shown in Figure 2, when TcCRT, N-TcCRT and HuCRT were compared in their capacities to inhibit morphogenesis, only the parasite-derived molecules significantly interfered with this process. The relevant TcCRT aminoacid sequence spans residues 20–191, corresponding to N-TcCRT. R-TcCRT did not affect capillary morphogenesis, in spite of its overlapping with N-TcCRT in aminoacids 136–191.

Chemotaxis is an essential step in capillary morphogenesis and angiogenesis. In HUVECs and Eahy926 cells, migration was inhibited in a dose-dependent manner by TcCRT (Figure 3). Cell migration inhibition by TcCRT may explain (at least partly) its potent effects on *in vitro* capillary morphogenesis and *ex vivo* capillary formation. These results agree with those describing the HuCRT capacity to increase cell binding to extracellular matrix, with consequent cell migration inhibition [28], [29].

As shown in Figure 4, TcCRT and N-TcCRT share the HuCRT capacity to specifically inhibit endothelial cell proliferation, a key initial event in angiogenesis [10]. These effects were not observed in a different cell line, like fibroblasts, used as negative controls. In HuCRT, the smallest anti-proliferative fragment spans aa 120–180 [10]. Since, as observed in the morphogenesis assay, R-TcCRT had no significant effect on HUVECs proliferation, relevant residues also map between aa 20–135. TcCRT interferes with pro angiogenic bFGF (Figure 4C), by unknown mechanisms. HuCRT also inhibits the proliferation of endothelial cells from diverse origins, such as FBHE [10], BAECs [30], HUVECs [31] and ECV304 [32], in response to bFGF and VEGF. R-TcCRT did not affect HUVECs proliferation (Figure 4D), nor morphogenesis (Figure 2F–H).

HUVECs proliferation inhibition by TcCRT may imply its involvement in the cell cycle or, alternatively, in cell death induction. TcCRT added at different concentrations to 24, 72 and 96 h HUVECs cultures did not induce apoptosis. Therefore, in the TcCRT-mediated inhibition of cell proliferation, a cytostatic effect, rather than apoptosis induction, may be mediated by the parasite molecule.

Recombinant proteins from *E. coli*, are normally contaminated with LPS, an antiangiogenic molecule [33]. In all the experiments discussed above, LPS was ineffective at concentrations equivalent to those present in the recombinant TcCRT preparations.

Although both HuCRT and TcCRT bind laminin, only the former interferes with endothelial cell adhesion and, as a consequence, with angiogenesis. Thus, the antiangiogenic TcCRT effects could be explained by other mechanisms, such as direct TcCRT interaction with endothelial cells. Alternatively, TcCRT could be internalized and fulfill other functions in the intracellular compartments. We now show that TcCRT binds to endothelial cells, followed by internalization. The transduction pathways involved are unknown. SREC-I (scavenger receptor expressed by endothelial cell-I) could be involved in these phenomena. HuCRT binds SREC-I, is endocytosed, and delivers associated peptides for cross presentation via MHC- I [34], [27], a fact compatible with our observations on the fucoidin (a specific SREC-I ligand [26], [27]) capacity to inhibit TcCRT internalization by HUVECs (Figure 5E). Besides being an endocytic receptor, SREC-I is an interesting candidate for signal transduction. Its intracellular domain comprises almost half of the molecule, surprisingly large among known scavenger receptors. It also contains several potential phosphorylation consensus sites [35], [36]. These results are compatible with the possibility that TcCRT internalization is a requisite to mediate its antiangiogenic effects on endothelial cells. Whether TcCRT interferes with the endothelial cell cytoskeleton, is unknown.

Perhaps, the parasite ability to inhibit angiogenesis interferes with immune/inflammatory responses against this aggressor. On the other hand, the role of angiogenesis in solid tumor progression has been long established in a variety of experimental models [37]. For six decades now, several reports have proposed a possible growth inhibitory effects that several *T. cruzi* strains may have on multiple transplanted and spontaneous tumors, in animals and humans [16], [17], [38]. The induction of specific immune anti-tumoral responses [39] and/or the secretion of “toxic substances” by the parasite [16], [40] were invoked to explain these effects, but no experimental evidences have been provided. Maybe, TcCRT, by interacting with endothelial cells and preventing neoangiogenesis, interferes in tumor growth and metastasis. For these reasons we tested the TcCRT and Hu-CRT capacity to inhibit *in vivo* the growth of a murine mammary tumor (TA3 MTXR). Only TcCRT displayed significant anti-tumor effects in both experiments. Moreover, the parasite molecule displayed stronger effects than HuCRT. Although maximum efforts were made to perform the experiments under similar conditions, the tumor growth was different by about 2-fold, in the experiments shown in Figure 6 A–B. The cell line is maintained in our laboratories, as ascites tumor in A/J mice, with weekly passages and the experiments were performed six months apart. Thus, although the conclusions drawn from both experiments are basically the same, we cannot rule out minor variations in handling, site of inoculation or in the cell line itself that could explain the different overall tumor growth observed in both experiments.

While the prevalence of tumor aggressions in wild and domestic *T. cruzi* hosts has not been assessed, in humans they may reach almost epidemic dimensions (*i.e.* mammary, prostate, ovary and cervix-uterine cancers, taken altogether). Thus, the TcCRT capacity to delay tumor growth, together with its anti inflammatory properties (derived from its complement inhibition capacity), may represent an evolutionary parasite adaptation, with final increased infectivity.

In synthesis, in this report we show that *T. cruzi* calreticulin has potent antiangiogenic activities, both on rat arterial (aortic ring assay) and human venous (HUVECs) endothelial cells. These properties map to the N-TcCRT domain in the parasite molecule. TcCRT plays key *in vitro* antiangiogenic roles, expressed as inhibition of capillary morphogenesis, proliferation and migration of endothelial cells. TcCRT internalization by endothelial cells is perhaps necessary in the antiangiogenic process. These facts, together with those previously reported by us, showing that TcCRT is a potent *in vivo* inhibitor of angiogenesis in a third vertebrate species (CAM assay), allow us to propose that the TcCRT antiangiogenic effects may be implicated in inflammatory and antineoplastic effects, with benefits for the parasite in its interactions with the vertebrate host. These findings may open interesting possibilities for the development of new antineoplastic strategies, especially if we consider that the parasite molecule displays stronger antiangiogenic and anti-tumor effects than its human counterpart. Biotechnological implications of these findings may be envisaged. Whether the antiangiogenic properties were consolidated, first in the parasite chaperone molecule, and HuCRT conserved some of these properties, as an evolutionary relict or, alternatively, the parasite hijacked this activity from its vertebrate host, remains an open question.

## Supporting Information

Alternative Language Abstract S1 Translation of the abstract into Spanish by Arturo Ferreira
(0.02 MB DOC)
