# Supplementary material for: Antiangiogenic and Antitumor Effects of Trypanosoma cruzi Calreticulin
Source: PLoS Negl Trop Dis. 2010 Jul 6;4(7):e730. doi: 10.1371/journal.pntd.0000730 (PMC2897838; doi:10.1371/journal.pntd.0000730)
Supplement: Alternative Language Abstract S1 — Translation of the abstract into Spanish by Arturo Ferreira (0.02 MB DOC) [file pntd.0000730.s001.doc]

**Resumen**

En América Latina, 18 millones de personas están infectadas con *Trypanosoma cruzi*, un protozoo que causa la enfermedad de Chagas. Las calreticulinas (CRT) de vertebrados son proteínas chaperonas multifuncionales intra y extracelulares, que unen calcio. Dado que la CRT humana (HuCRT) inhibe el crecimiento capilar (angiogénesis) y suprime el crecimiento tumoral, la presencia de estas funciones en *T. cruzi* CRT (TcCRT) puede tener consecuencias interesantes en las interacciones hospedero/parásito. Previamente, hemos clonado y expresado TcCRT y demostrado que, cuando se transloca desde el retículo endoplásmico al área de emergencia flagelar de los tripomastigotes, promueve la infectividad, inactiva al sistema del complemento, un brazo de defensa innata, e inhibe la angiogénesis en la membrana corioalantoídea de huevos de gallina. Ahora mostramos que TcCRT inhibe la angiogénesis porque interfiere con la multiplicación de células endoteliales, con la migración y morfogénesis capilar *in vitro,* y con la angiogénesis en anillos aórticos de rata. La molécula parasitaria también despliega importantes efectos antitumorales. En estas actividades, TcCRT es más efectiva que la contraparte humana. Quizás, porque inhibe la angiogénesis, TcCRT es anti inflamatoria, dificultando así la respuesta inmune antiparasitaria. El efecto antiangiogénico de TcCRT podría explicar, al menos parcialmente, su efecto antitumoral, descrito aquí y las propiedades antitumorales propuestas por otros laboratorios para la infección con *T. cruzi*.
